# MLSolvA: solvation free energy prediction from pairwise atomistic interactions by machine learning

**DOI:** 10.1186/s13321-021-00533-z

**Published:** 2021-07-31

**Authors:** Hyuntae Lim, YounJoon Jung

**Affiliations:** Department of Chemistry, Seoul National University, Seoul, 08826 South Korea

## Abstract

Recent advances in machine learning technologies and their applications have led to the development of diverse structure–property relationship models for crucial chemical properties. The solvation free energy is one of them. Here, we introduce a novel ML-based solvation model, which calculates the solvation energy from pairwise atomistic interactions. The novelty of the proposed model consists of a simple architecture: two encoding functions extract atomic feature vectors from the given chemical structure, while the inner product between the two atomistic feature vectors calculates their interactions. The results of 6239 experimental measurements achieve outstanding performance and transferability for enlarging training data owing to its solvent-non-specific nature. An analysis of the interaction map shows that our model has significant potential for producing group contributions on the solvation energy, which indicates that the model provides not only predictions of target properties but also more detailed physicochemical insights.

**Supplementary Information:**

The online version contains supplementary material available at 10.1186/s13321-021-00533-z.

## Introduction

The importance of solvation or hydration mechanisms and their accompanying free energy change has rendered in silico calculation methods for the solvation energy one of the most important applications in computational chemistry [3, 7, 12, 13, 15, 16, 18, 19, 21, 23, 33–37, 39, 40, 43, 44, 50, 52, 54, 57, 58, 65, 67, 71, 73, 79, 81]. Solvation free energy directly influences numerous chemical properties in condensed phases and plays a dominant role in various chemical reactions, such as drug delivery [18, 21, 51, 67], organic synthesis [53], electrochemical redox reactions [1, 30, 47, 72], etc.

Atomistic computer simulation approaches directly provide the microscopic structure of the solvent shell, which surrounds solute molecules [12, 21, 27, 36, 65, 81]. The solvation shell structure offers detailed physicochemical information, such as microscopic mechanisms on solvation or the interplay between the solvent and the solute molecules when using an appropriate force field and molecular dynamics parameters. However, the *explicit solvation* methods mentioned above require extensive numerical calculations as each individual molecular system must be simulated. Practical problems in the explicit solvation model restrict its applications to simulations of classical molecular mechanics [12, 65, 81] or to a limited number of QM/MM approaches [27, 36].

In classical mechanics approaches for macromolecules or calculations for small compounds at the quantum-mechanical level, the concept of *implicit solvation* enables calculation of the solvation free energy with feasible time and computational costs when one considers a given solvent as a continuous and isotropic medium, whose behavior is described by the Poisson–Boltzmann equation [16, 23, 33–35, 39, 40, 43, 54, 73]. Numerous theoretical advances have been made to construct the continuum solvation model, which involves parameterized solvent properties: the polarizable continuum model (PCM) [43], the conductor-like screening model (COSMO) [35] and its variations [32, 34], generalized Born approximations, such as solvation model based on density (SMD) [39] or solvation model 6, 8, 12, etc. (SMx) [16, 40].

The structure–property relationship (SPR) via machine learning is rather a novel approach, which predicts the solvation free energy from a completely different point of view compared to computer simulation approaches with precisely defined theoretical backgrounds [11, 74]. Although we may not expect to obtain detailed chemical or physical insights other than the target property because this is a regression analysis in its nature, SPR has demonstrated significant potential in terms of transferability and outstanding computational efficiency [11, 74, 79]. Recent progress in machine learning (ML) techniques [59] and their implementation in computational chemistry [8, 79] are currently promoting broad applications of SPR in numerous chemical studies [1–4, 7, 9, 10, 15, 18, 20, 26, 28, 29, 50–52, 55, 56, 58, 60–62, 64, 66, 68, 71, 75–78, 82]. These studies show that ML guarantees faster calculations than computer simulations and more precise estimations than traditional SPR estimations; a considerable number of models showed accuracies comparable to ab initio solvation models in the aqueous system [79].

Previously, we introduced a novel artificial neural-network-based ML solvation model called *Delfos*, which predicts free energies of solvation for generic organic solvents [37]. The model not only has a significant potential for showing an accuracy comparable to the state-of-the-art computational chemistry methods [33, 40], but also offers information by which substructures play a dominant role in the solvation process. Herein we propose a novel approach to the ML model for the solvation energy estimation called *MLSolvA*, which is based on the group-contribution method. The key idea of the proposed model is the calculation of pairwise atomic interactions by mapping them into inner products of atomic feature vectors, while each encoder network for the solvent and the solute extracts such atomic features. We believe that the proposed approach presents a powerful tool for understanding solvation processes and is capable of strengthening various solvation models via computer simulations.

The paper is constructed as follows: in “Methods” section, we introduce the theoretical background of applied ML techniques and the overall architecture of our proposed model. “Results and discussion” section quantifies the model’s prediction performance with 6239 data points, mainly focusing on pairwise atomic interactions and corresponding group contributions on the solvation free energy. “Conclusions” section summarizes and concludes our work.

## Methods

### Model architecture

In the proposed model, the linear regression task of calculating the solvation free energy between the given solvent and solute molecules starts with embedded atomistic vector representations [25, 37] of the solvent molecule consisting of $\mathbf {x}_{\alpha }$’s and the solute molecule consisting of $\mathbf {y}_{\beta }$’s, where $\alpha$ and $\beta$ are the atom indices. Then we can describe the given molecule as a tensor, which is a collection (or a sequence) of atomistic vectors:

1a $$\begin{aligned} \mathbf {X}&=\left\{ \mathbf {x}_{\alpha } \right\} \quad \alpha \in \{1, \ldots , M_{a}\}, \end{aligned}$$

1b $$\begin{aligned} \mathbf {Y}&=\left\{ \mathbf {y}_{\beta } \right\} \quad \beta \in \{1, \ldots , M_{b}\}, \end{aligned}$$

where $\mathbf {x}_{\alpha }$ and $\mathbf {y}_{\beta }$ are the $\alpha$-th row of $\mathbf {X}$ and the $\beta$-th row of $\mathbf {Y}$, respectively. Here, dimensions of two tensors are $M_{a} \times D$ for $\mathbf {X}$ and $M_{b} \times D$ for $\mathbf {Y}$, where $M_{a}$ and $M_{b}$ are the sizes of the given solvent and solute (by heavy atom count), and *D* is the embedding dimension. Then, the encoder function learns of their chemical structures and extracts feature tensors $\mathbf {P}$ for the solvent and $\mathbf {Q}$ for the solute,

2a $$\begin{aligned} \mathbf {P}&=\left\{ \mathbf {p}_{\alpha } \right\} = \mathrm {Encoder} (\mathbf {X}), \end{aligned}$$

2b $$\begin{aligned} \mathbf {Q}&=\left\{ \mathbf {q}_{\beta } \right\} = \mathrm {Encoder} (\mathbf {Y}). \end{aligned}$$

Dimensions of $\mathbf {P}$ and $\mathbf {Q}$ are $M_{a} \times N$ and $M_{b} \times N$, respectively. The numbers of rows are invariable because the encoder function should preserve the topological structure of the given molecule, however, the column dimension, *D* can differ with *N*, depending on the number of hidden units of the encoder. Rows of $\mathbf {P}$ and $\mathbf {Q}$, $\mathbf {p}_{\alpha }$ and $\mathbf {q}_{\beta }$ involve atomistic chemical features of atoms $\alpha$ and $\beta$, which are directly related to the target property, i.e. the solvation free energy in the present work. We calculate the un-normalized attention score (or *chemical similarity*) between the atoms $\alpha$ and $\beta$ with Luong’s dot-product attention [38],

3 $$\begin{aligned} \mathbf {I}_{\alpha \beta } = - \mathbf {p}_{\alpha } \cdot \mathbf {q}_{\beta }, \end{aligned}$$

which is an element of $M_{a} \times M_{b}$ tensor of atomistic interactions, $\mathbf {I}$. Because our target quantity is the free energy of solvation, we expect such chemical similarity $\mathbf {I}_{\alpha \beta }$ to correspond to atomistic interactions between $\alpha$ and $\beta$, which includes both energetic and entropic contributions. Eventually, the free energy of solvation of the given solvent–solute pair, which is the final regression target, is expressed as a simple summation of atomistic interactions:

4 $$\Delta G_{\mathrm sol}^{\circ } = \sum _{\alpha =1}^{M_{a}}\sum _{\beta =1}^{M_{b}} {\mathbf I}_{\alpha \beta }.$$

Certainly, one can also calculate the free energies of solvation from two molecular feature vectors, which represent the solvent properties $\mathbf {u}$ and the solute properties $\mathbf {v}$, respectively:

5 $$\Delta G_{\mathrm sol}^{\circ } = {\mathbf u} \cdot {\mathbf v} = \left( \sum _{\alpha =1}^{M_{a}} {\mathbf p}_{\alpha } \right) \cdot \left( \sum _{\beta =1}^{M_{b}} {\mathbf q}_{\beta } \right) .$$

**Fig. 1:**
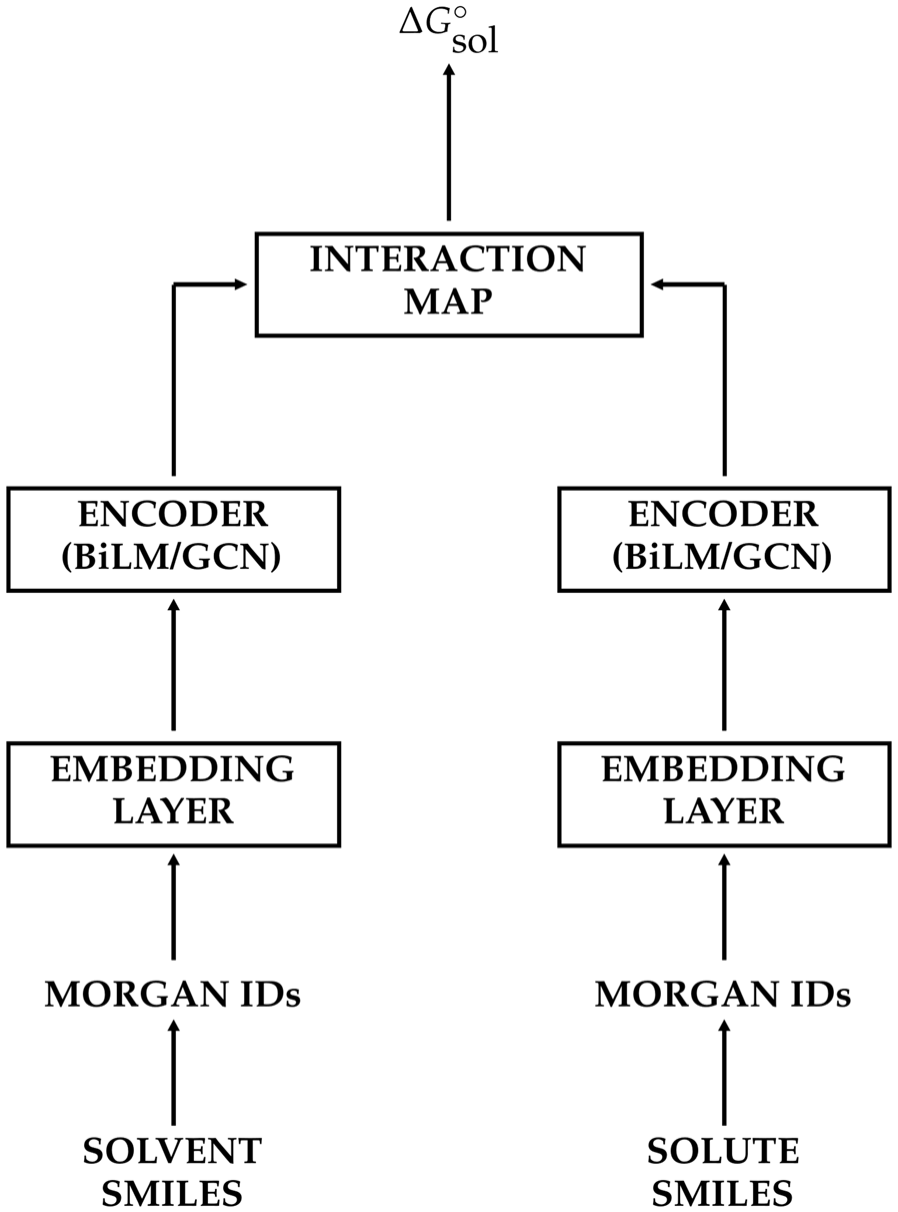
Schematic of MLSolvA architecture. Each encoder network extracts atomistic feature vectors given pre-trained vector representations, and the interaction map calculates pairwise atomistic interactions from Luong’s dot-product attention [38]

The inner-product relation between molecular feature vectors $\mathbf {u}$ and $\mathbf {v}$ has a formal analogy with the solvent-gas partition coefficient calculation method via the solvation descriptor approach [63, 70]. Figure 1 illustrates an overview of the architecture of the proposed ML solvation model.

### Encoder networks

We chose and compared two different neural network models to encode the input molecular structure and extract important structural or chemical features that are strongly related to solvation behavior. One is the bidirectional language model (BiLM) [49] based on the recurrent neural network (RNN), and the other is the graph convolutional neural network (GCN) [31] which explicitly handles the connectivity (bonding) between atoms with the adjacency matrix.

The detailed mathematical expressions of the BiLM, which is the first model, are given as follows [49]:

6a $$\begin{aligned} \overrightarrow{\mathbf {H}}^{(i + 1)}&=\overrightarrow{\mathrm {RNN}} (\overrightarrow{\mathbf {H}}^{(i)}), \end{aligned}$$

6b $$\begin{aligned} \overleftarrow{\mathbf {H}}^{(i + 1)}&= \overleftarrow{\mathrm {RNN}} (\overleftarrow{\mathbf {H}}^{(i)}). \end{aligned}$$

In Eq. 6, the right-headed arrow in $\overrightarrow{\mathrm {RNN}}$ denotes a forward-directed recurrent unit that propagates from the leftmost to the rightmost sequence. The BiLM likewise involves a backward-directed recurrent neural network ($\overleftarrow{\mathrm {RNN}}$) and propagates from the rightmost to the leftmost sequence as well. The superscript (*i*) in hidden layers $\mathbf {H}^{(i)}$ denotes the position at the stacked configuration. In the first layer, both forward and backward-directed RNN share the pre-trained sequence $\mathbf {X}$ as an input, $\overrightarrow{\mathbf {H}}^{(0)} =\overleftarrow{\mathbf {H}}^{(0)} = \mathbf {X}$.

Furthermore, more improved versions of RNNs, such as the gated recurrent unit (GRU) [14] or the long-short term memory (LSTM) [24] are more suitable when we consider cumulated numerical errors due to the deep-structured nature of RNNs [5],

7 $$\begin{aligned} \mathbf {H}^{(i)} = \overrightarrow{\mathbf {H}}^{(i)} + \overleftarrow{\mathbf {H}}^{(i)}. \end{aligned}$$

Hidden layers from the forward and backward RNNs are then merged into a single sequence, as described in Eq. 7. Finally, we obtain the sequence of chemical feature vectors of the $\alpha$-th atom in the given solvent with weighted summation of stacked RNN layers,

8 $$\begin{aligned} \mathbf {P} = \sum _{i} c_{i} \mathbf {H}^{(i)}, \end{aligned}$$

where each weighing factor $c_{i}$ is also a trainable parameter. The encoder function for solutes has an identical neural network architecture, which converts the pre-trained solute sequence $\mathbf {Y}$ into the feature sequence $\mathbf {Q}$. In addition, each layer in the encoder must share the same number of hidden units *N* due to Eqs. 3, 8.

We consider the graph convolutional neural network (GCN), which is one of the most well-known algorithms in the chemical applications of neural networks [29, 31]. The GCN model represents the input molecule as a mathematical graph, instead of a simple sequence: each node corresponds to the atom, and each edge in the adjacency matrix $\mathbf {A}$ involves connectivity (or existence of bonding) between atoms:

9 $$\begin{aligned} \mathbf {H}^{(i + 1)} = \mathrm {GCN} ( \mathbf {H}^{(i)}, \mathbf {A} ). \end{aligned}$$

The role of the adjacency matrix in the GCN constrains convolution filters to the node itself and its nearest neighbors. Equation 10 describes a more detailed mathematical expression of the skip-connected GCN [31]:

10 $$\begin{aligned} \mathrm {GCN} (\mathbf {H}, \mathbf {A}) = \sigma \left(\mathbf {D}^{-1/2} \mathbf {A} \mathbf {D}^{-1/2} \mathbf {H} \mathbf {W}_{1} +\mathbf {H} \mathbf {W}_{2} + \mathbf {b}\right), \end{aligned}$$

where $\mathbf {D}$ is the degree matrix, $\mathbf {W}_{1}$ and $\mathbf {W}_{2}$ are convolution filters, $\mathbf {b}$ is the bias vector, and $\sigma$ denotes the activation function chosen as the hyperbolic tangent in the proposed model. The GCN encoder includes the stacked structure, and we obtain the feature sequence for each molecule in the same manner as described in Eq. 8.

## Results and discussion

### Computational setup and results

For the training and tests of the proposed neural network, we prepared 6239 experimental measures of free energies of solvation for 935 organic solvents and 146 organic solutes. A total of 642 experimentally measured values of the free energy of hydration are collected from the FreeSolv database [44, 45], and 5597 data points for non-aqueous solvents were collected with the Solv@TUM database version 1.0 [22, 23]. The compounds in the dataset comprise ten different kinds of atoms that are common in organic chemistry, viz. hydrogen, carbon, oxygen, sulfur, nitrogen, phosphorus, fluorine, chlorine, bromine, and iodine. The maximum heavy-atom count is 28 for the solute molecules and 18 for the solvent molecules.

At the very first stage, we perform the skip-gram pretraining process for 10,229,472 organic compounds, which are collected from the ZINC15 database [69], with Gensim 3.8.1 and Mol2Vec skip-gram model to construct the 128-dimensional embedding lookup table [25]. A total of 634 solutes 120 solvents in the FreeSolv/Solv@TUM combined dataset appear in the pretraining dataset. The pretraining process generates atomistic vector representations of the heavy atoms in different chemical environments distinguished by the Morgan identifiers [25, 46]. Although the skip-gram task does not guarantee a significant enhancement of the model’s accuracy, we found that the pretrained model yielded more stable results in terms of RMSE variance (Additional file 1: Table S2). For the implementation of the neural network model, we mainly use TensorFlow 2.5.0 framework [41]. Each model has L2 regularization to prevent excessive changes on weights and to minimize the variance, and uses the RMSprop algorithm for minimization:

11a $$\begin{aligned} G_{t}&=\rho G_{t - 1} + (1 - \rho )(\nabla _{w} L_{t})^{2}, \end{aligned}$$

11b $$\begin{aligned} w_{t}&=w_{t - 1} + \frac{\eta }{\sqrt{G_{t} + \epsilon }} \nabla _{w} L_{t}, \end{aligned}$$

where $L_{t}$ is the loss function, chosen as the mean squared error (MSE) in this work. $G_{t}$ denotes a moving average of the squared gradient of $L_{t}$, and it scales update rates of the weight, *w*. The other parameters play the following roles: $\eta$ is the initial learning rate, $\rho$ is a discounting factor for the moving average, and $\epsilon$ prevents possible bursting of $1/\sqrt{G_{t}}$ for numerical stability. The selection of the optimized model for the target property is realized by an extensive Bayesian optimization process for tuning model hyperparameters [6] (Additional file 1: Table S1)

**Fig. 2:**
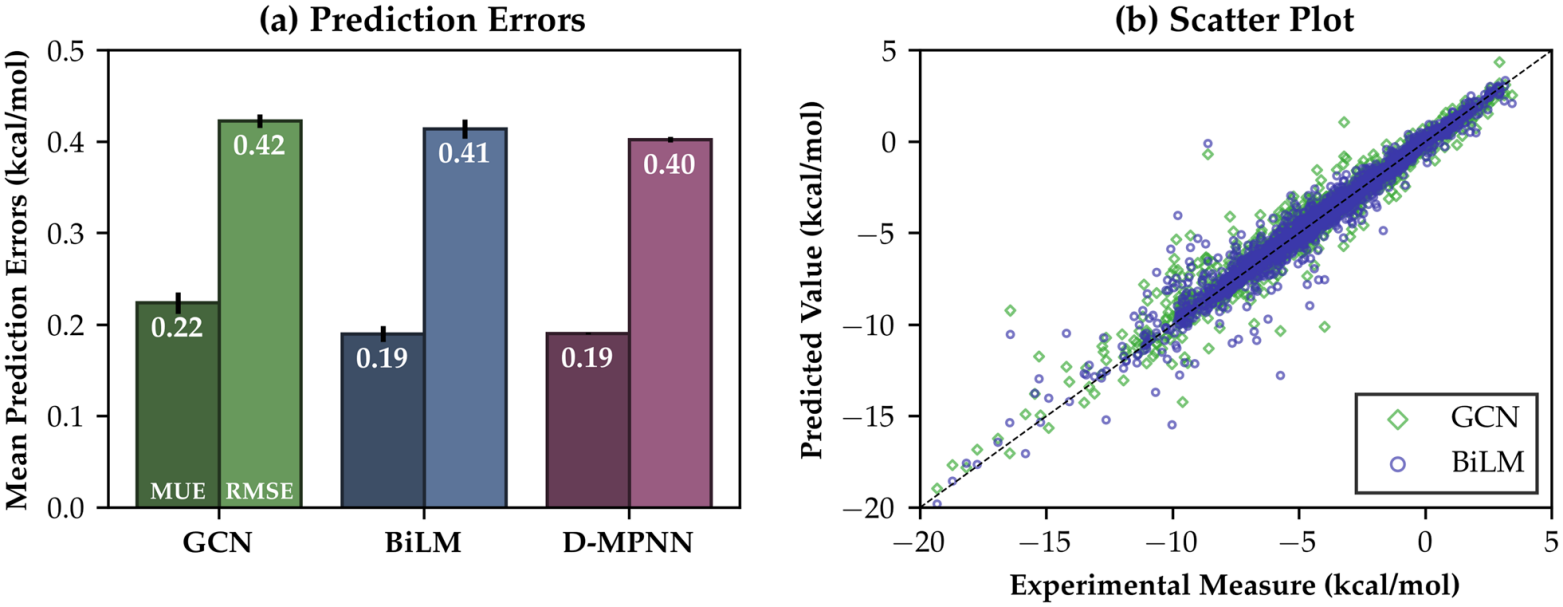
**a** Prediction errors for BiLM and GCN models in kcal/mol, obtained by five-fold nested cross validation results. Results taken from the D-MPNN model [17, 80] are also depicted for comparison. **b** Scatter plot between experimental values and predicted values by the models. Green circles depict the BiLM model, while the GCN results are depicted by blue circles

We employ five-fold nested cross-validation (CV) to evaluate the prediction accuracy of the chosen model. Nested CV incorporates two CV loops: the inner loop selects the best hyperparameters over the validation set, while the outer loop evaluates the model’s final performance of prediction. This procedure prevents overlap and possible *information leakage* between the validation and test sets [48]. To evaluate the uncertainty of results taken from CV tasks, we take averages for all mean errors over eight independent nested CV runs, split from different random states. The results for the test run using nested CV tasks for the optimized models are shown in Fig. 2. We found that the BiLM encoder with LSTM layer performs slightly better than the GCN encoder, although their differences are not pronounced. The mean unsigned prediction error (MUE) for the BiLM/LSTM encoder model is 0.19 kcal/mol, while the GCN model results in MUE = 0.22 kcal/mol. Both values show that the proposed mechanism works efficiently and guarantees excellent prediction accuracies for well-trained chemical structures. We also perform the same CV procedure using the Direct Message-Passing Neural Network (D-MPNN) model [17], which is available at the chemprop package [80]. The prediction error of the D-MPNN model on the same dataset is MUE = 0.19 kcal/mol, which indicates our proposed model design yields a comparable accuracy with the deep-learning model in the current state-of-the-art (see Additional file 2 for the raw data).

### Visualization of chemical similarity

**Fig. 3:**
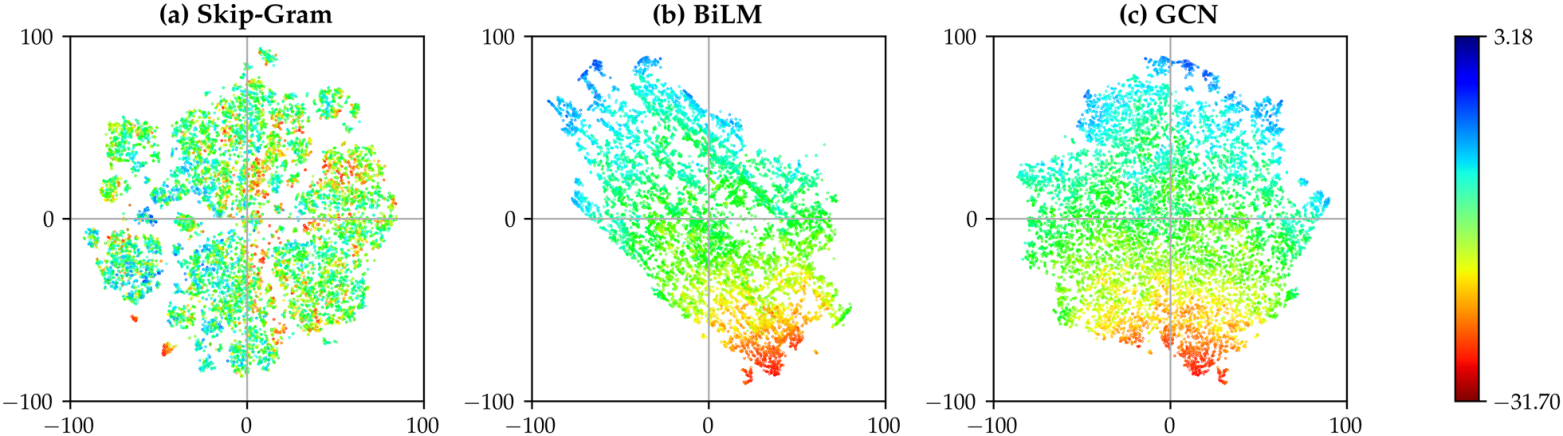
Two-dimensional visualizations on **a** pre-trained vector from the skip-gram model $\sum _{\beta } \mathbf {y}_{\beta }$ and **b**, **c** extracted molecular feature vector $\mathbf {v}$ for 15,432 solutes. We reduce the dimensions of each vector using the t-SNE algorithm. The color representation denotes the hydration energy of each point

The fundamental idea behind the proposed model is the encoder network, which maps complex chemical features into a vector representation. Because we aim for the free energy of solvation as the target property, geometries in the vector space must have a strong correlation with their solvation properties. We validate this point with t-Stochastic Neighbor Embedding (t-SNE) visualizations for pre-trained solute vectors $\mathbf {y}$, and encoded molecular features $\mathbf {v}$ [48, 56]. The dimensions of those vectors must be reduced for visualization, because we use 128-dimensional vector representations, which cannot be directly drawn into a graph. Figure 3 presents the reduced geometries of $\mathbf {y}$ and $\mathbf {v}$ in two-dimensional space, which indicates that the proposed encoder neural network works as intended. Color shading depicts the predicted hydration free energies for 15,432 points, whose structures are randomly taken from the ZINC15 [69]; red and blue dots correspond to low and high hydration free energy cases, respectively. The significant correlation between reduced molecular feature vectors and predicted free energy values indicates how the proposed architecture extracts important molecular features and makes the prediction from them. Meanwhile, the pre-trained solute vectors from the skip-gram embedding model exhibit only weak correlations.

### Advantage of model: transferability

**Fig. 4:**
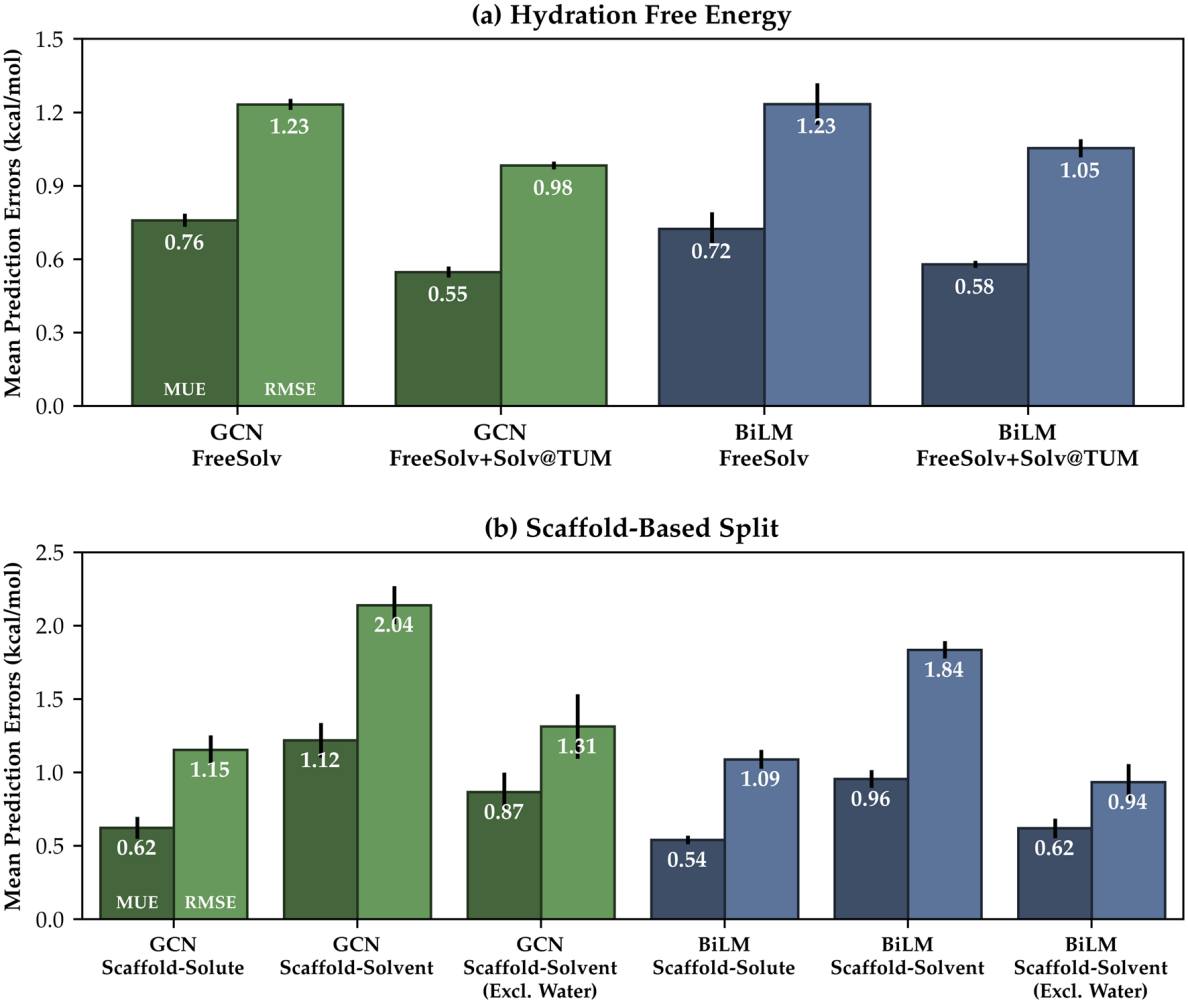
**a** CV-results for FreeSolv hydration energies with two different training datasets. Deep-colored boxes depict CV results with augmented dataset with Solv@TUM database. **b** CV results for two scaffold-based split methods using K-means clustering algorithm

Because our proposed neural network model is solvent-generic, as it considers both the solvent and solute structures as separate inputs, it exhibits a distinct and advantageous character when compared to other solvent-specific ML solvation models. Let us consider the following possible situation where one wants to predict the solvation free energy of a solute compound ${\mathcal {A}}$ in a solvent ${\mathcal {X}}$, $\Delta G^{\circ }_{{\mathcal {A}}{\mathcal {X}}}$. Since the model has been trained with varied kinds of solvents and solutes, the training database will likely involve solvation free energy measures for *A* in other different solvents, e.g. $\Delta G^{\circ }_{{\mathcal {A}}{\mathcal {Y}}}$, $\Delta G^{\circ }_{{\mathcal {A}}{\mathcal {Z}}}$, and so on. Then the model would have already become aware of the structural features of ${\mathcal {A}}$, which could help the prediction of $\Delta G^{\circ }_{{\mathcal {A}}{\mathcal {X}}}$ [37]; this mechanism would not happen if the model supports only one kind of solvent. Therefore, one of the largest advantages of our model is that we can easily enlarge the dataset for training, even in the scenario where we want to predict solvation free energies for a specific solvent. Figure 4 shows five-fold CV results for 642 hydration free energies (FreeSolv) from both BiLM and GCN models in two different situations. One uses only the FreeSolv [44, 45] database for training and tests, whereas the other uses both the FreeSolv and the Solv@TUM [22, 23] databases. Although the Solv@TUM database only involves non-aqueous data points, it enhances each model’s accuracy by approximately 20% (BiLM) to 30% (GCN) in terms of the MUE. These results imply that there are possible applications of transfer learning to other solvation-related properties, such as aqueous solubilities [18] or octanol–water partition coefficients.

However, in some other situations, one may be concerned that the repetitive training for a single compound may cause overfitting by the model, and they could weaken the predictivity for the structurally new compound, which is considered an extrapolation. We investigate the model’s predictivity for extrapolation situations with a *scaffold-based* split [20, 37, 42, 77]. Instead of the ordinary K-fold CV task with the random and uniform split method, the K-means clustering algorithm builds each fold with the Molecular ACCess system (MACCS) substructural fingerprint [77]. An extreme extrapolation situation can be simulated through CV tasks over the folds, which are constructed by the clustering on solvents or solutes. As shown in Fig. 4, the scaffold-based split on the solvents shows more degradation of prediction performances than the scaffold-solute-based split due to the limited kinds of solvent compounds in the dataset, although both results are still within an acceptable error level, given chemical accuracy of ~ 1.0 kcal/mol (raw data is available in Additional file 2). A considerable part of degradation in the scaffold-solvent-based split arises from water solvent due to its unusually distinct physicochemical nature from other organic solvents [37]. Furthermore, the embedding scheme we use generates a unique Morgan identifier for the oxygen of water (864666390), which cannot be recognized or trained from the other hydroxyl oxygens such as alcohols (864662311).

### Group contributions

Although we showed that the proposed NN model guarantees an excellent predictivity for solvation energies of various solute and solvent pairs, the main objective of the present study is to obtain the solvation free energy as the sum of decomposed interatomic interactions, as described in Eqs. 3 and 4. To verify the feasibility of the model’s solvation energy estimation to decompose into group contributions, we define the sum of atomic interactions $\mathbf {I}_{\alpha \beta }$ over the solvent indices $\alpha$ as the group contributions of the $\beta$-th solute atom:

12 $$\begin{aligned} \mathbf {I}_{\beta } = \sum _{\alpha } \mathbf {I}_{\alpha \beta } \end{aligned}.$$

**Fig. 5:**
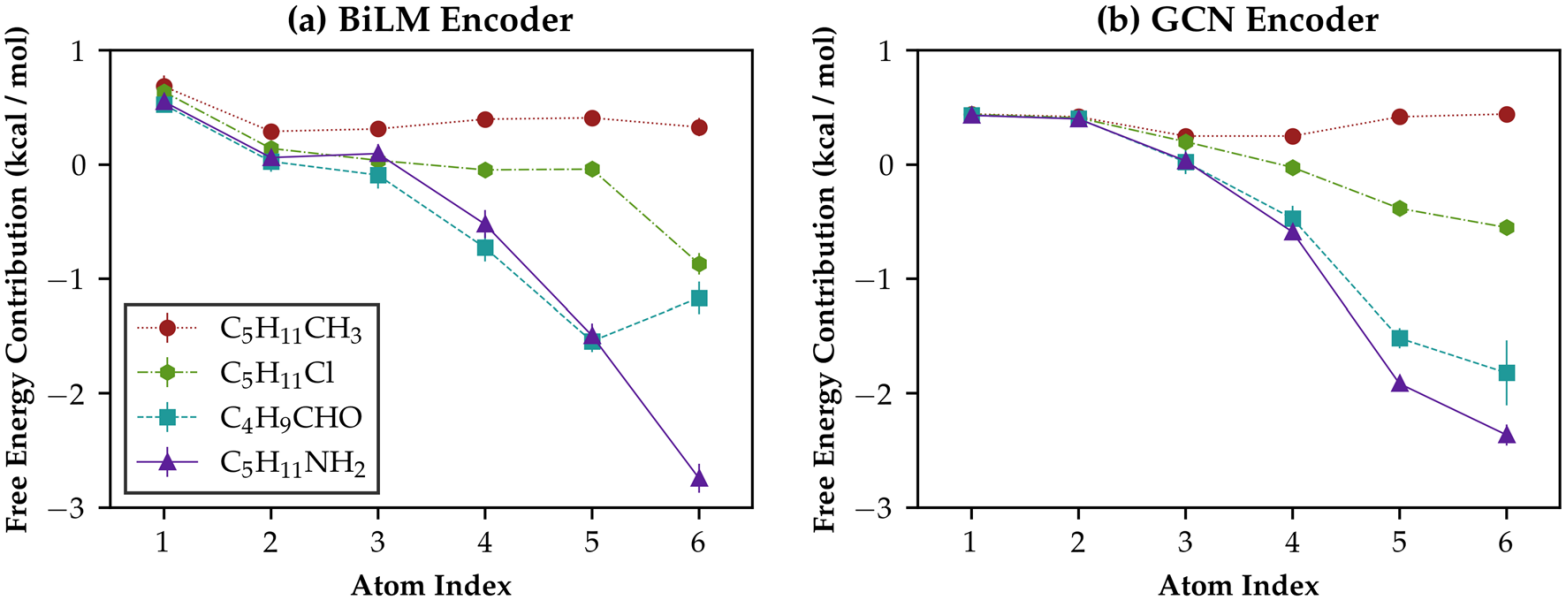
ML-calculated atomistic group contributions for five small organic compounds with six heavy atoms (excluding the hydrogens). The atom index starts from the left-most point of the given molecule and only counts heavy atoms

Figure 5 shows hydration free energy contributions for five small organic solutes with six heavy atoms: *n*-hexane, 1-chloropentane, pentaldehyde, 1-aminopentane, and benzene. Both the BiLM and the GCN model exhibit a similar tendency in group contributions; the model estimates that atomic interactions between the solute atoms and water increase near the hydrophilic groups. It is obvious that each atom in benzene must have identical contributions to the free energy; however, the results in Fig. 5 clearly show that the BiLM model makes faulty predictions while the GCN model works well as expected. We believe that this malfunctioning of the BiLM model originates from the sequential nature of the recurrent neural network. Because the RNN considers that the input molecule is only a simple sequence of atomic vectors, and there are no explicit statements that involve bonding information, the model is not aware of the cyclic shape of the input compound [29, 51]. We conclude that it is inevitable to use explicitly bound (or connectivity) information when constructing a group-contribution based ML model, even though the RNN-based model provides good predictions in terms of their sum.

## Conclusions

We introduced a novel approach for ML-based solvation energy prediction, which exhibits great potential to provide physicochemical insight on the solvation process. The novelty in our neural network model lies in the ability to calculate pairwise atomic interactions from the inner products of atomistic feature vectors [38]. This idea gives us more straightforward and interpretable information on intermolecular interactions between the solute and solvent molecules, and the model calculates the solvation free energy from the group-contribution-based prediction.

We quantified the proposed model’s prediction performances for 6293 experimental data points of solvation energies, which were taken from the FreeSolv [44, 45] and Solv@TUM [22, 23] databases. We found a significant geometrical correlation between molecular feature vectors and predicted properties, which confirms that the proposed model successfully extracts chemical properties and maps them into vector representations. The estimated prediction MUEs from K-fold CV are 0.19 kcal/mol for the BiLM encoder and 0.23 kcal/mol for the GCN model.

The K-fold CV results from the scaffold-based split [77] showed that the prediction accuracy decreases by three times in extreme extrapolation situations; however, they nevertheless exhibit moderate performances, which was MUE = 0.60 kcal/mol. Moreover, we found that the solvent-generic structure of the proposed model is appropriate for enlarging the dataset size, i.e. experimental data points for a particular solvent are transferable to other solvents. We conclude that this transferability is the reason for our model’s outstanding predictivity [37].

Finally, we examined pairwise atomic interactions obtained from the interaction map and found a clear tendency between hydrophilic groups and their contributions to the hydration free energy. Such results are obtained from a simple, graph-convolution based neural network instead of deep learning models in the current state-of-the-art [20, 62]. Despite the limitation of a simple model, the model showed a reliable performance with the concept of group contributions approach via neural networks. Thus, we expect that the suggested concept would have further developments with more progressed ML models or applications for molecular dynamics simulations [12, 13]. We believe that our model is capable of providing detailed information on the solvation mechanism, as well as the predicted value of the target property.

## Supplementary Information

**Additional file 1. **List of model hyperparameters for the Bayesian optimization process (Table S1) and influence of the pre-training task on the prediction results (Table S2). 

**Additional file 2. **Raw prediction results file for the random CV and scaffold-based CV tasks.

## Data Availability

The source code is available on GitHub repository: https://github.com/ht0620/mlsolva. The data supporting the findings of this study are available within the article and its additional information files.
