# Supplementary material for: MLSolvA: solvation free energy prediction from pairwise atomistic interactions by machine learning
Source: J Cheminform. 2021 Jul 31;13:56. doi: 10.1186/s13321-021-00533-z (PMC8325294; doi:10.1186/s13321-021-00533-z)
Supplement: Supplementary file 1 — Additional file 1. List of model hyperparameters for the Bayesian optimization process (Table S1) and influence of the pre-training task on the prediction results (Table S2). [file 13321_2021_533_MOESM1_ESM.pdf]

Electronic Supporting Information

MLSolvA: Solvation Free Energy Prediction from Pairwise Atomistic Interactions by Machine Learning

Hyuntae Lim and YounJoon Jung*

Department of Chemistry, Seoul National University, Seoul 08826, Korea

June 18, 2021

A. Model Optimization

We fix the embedding dimension and each layer's hidden units as 128 and use the RMSprop algorithm for the backpropagation process. Then we utilize the Bayesian optimization procedure[1] to optimize the other hyperparameters: L2 regularization, learning rate (η), discounting factor (ρ), model depth, batch size, and epochs. For the other adjustable parameters like ϵ in Eq. 11 or initial weights of layers, we use default values of Tensorflow 2.5.0[3]. The validation loop selects the best set of hyperparameters after 50 iterations, and their searching ranges are shown in Table S1. We use hyperopt library[1], which is available at https://github.com/hyperopt/hyperopt, to implement the optimization task.

B. Efficacy of Pretraining

Table S2 shows a comparative study on the effect of pretraining with eight different cross-validation tasks. The skip-gram pretraining task was executed with 10,229,472 organic com-

*yjjung@snu.ac.kr

Hyperparameter	Range	Distribution
L2 regularization	$10^{-6} \sim 10^{-2}$	Log uniform
Initial learning rate (η in Eq. 11)	$10^{-5} \sim 10^{-1}$	Log uniform
Discounting factor (ρ in Eq. 11)	$0.1 \sim 0.9$	Uniform
Model depth	2, 3, 4, 5, 6	
Batch size	32, 64, 96	
Epochs	50, 100, 150, 200	

Table S1: List of model hyperparameters and their ranges for the Bayesian optimization process.

	BiLM w/o Pretraining	BiLM Skip-gram	GCN w/o Pretraining	GCN Skip-gram
CV run #1	0.406	0.402	0.443	0.426
CV run #2	0.424	0.415	0.428	0.422
CV run #3	0.446	0.410	0.394	0.438
CV run #4	0.455	0.415	0.421	0.460
CV run #5	0.418	0.410	0.452	0.442
CV run #6	0.416	0.406	0.406	0.435
CV run #7	0.435	0.405	0.417	0.435
CV run #8	0.416	0.419	0.424	0.443
Avg.	0.427	0.410	0.423	0.438
Std. Err.	0.016	0.005	0.017	0.011

Table S2: Impact of skip-gram pretraining task for both BiLM and GCN models. All prediction errors are calculated in RMSE and kcal/mol.

pounds, which were collected from the ZINC15 database[4, 2], while we initialized all embedding weights to zero for models without pretraining. Further, we used trainable embedding layers for all four cases. All CV tasks of this section were done under the following set of hyperparameters:

- L2 regularization: 10^{-5}
- Initial learning rate: 10^{-3}
- Discounting factor: 0.9
- Model depth: 3 (BiLM), 4 (GCN)
- Batch size: 32
- Epochs: 100 (BiLM), 200 (GCN)

References

[1] Bergstra, J., Yamins, D., Cox, D.D.: Making a science of model search: hyperparameter optimization in hundreds of dimensions for vision architectures. In: Proceedings of the 30th International Conference on International Conference on Machine Learning - Volume 28, ICML'13, pp. I–115–I–123. JMLR.org, Atlanta, GA, USA (2013)

[2] Jaeger, S., Fulle, S., Turk, S.: Mol2vec: Unsupervised Machine Learning Approach with Chemical Intuition. Journal of Chemical Information and Modeling **58**(1), 27–35 (2018). DOI 10.1021/acs.jcim.7b00616. URL https://pubs.acs.org/doi/10.1021/acs.jcim.7b00616

[3] Martín Abadi, Ashish Agarwal, Paul Barham, Eugene Brevdo, Zhifeng Chen, Craig Citro, Greg S. Corrado, Andy Davis, Jeffrey Dean, Matthieu Devin, Sanjay Ghemawat, Ian Goodfellow, Andrew Harp, Geoffrey Irving, Michael Isard, Jia, Y., Rafal Jozefowicz, Lukasz Kaiser, Manjunath Kudlur, Josh Levenberg, Dan Mané, Rajat Monga, Sherry Moore, Derek Murray, Chris Olah, Mike Schuster, Jonathon Shlens, Benoit Steiner, Ilya Sutskever, Kunal Talwar, Paul Tucker, Vincent Vanhoucke, Vijay Vasudevan, Fernanda Viégas, Oriol Vinyals, Pete Warden, Martin Wattenberg, Martin Wicke, Yuan Yu, Xiaoqiang Zheng: TensorFlow: Large-Scale Machine Learning on Heterogeneous Systems (2015). URL http://tensorflow.org/

[4] Sterling, T., Irwin, J.J.: ZINC 15 – Ligand Discovery for Everyone. Journal of Chemical Information and Modeling **55**(11), 2324–2337 (2015). DOI 10.1021/acs.jcim.5b00559. URL https://pubs.acs.org/doi/10.1021/acs.jcim.5b00559
